# *De novo* Comparative Transcriptome Analysis of *Acremonium chrysogenum*: High-Yield and Wild-Type Strains of Cephalosporin C Producer

**DOI:** 10.1371/journal.pone.0104542

**Published:** 2014-08-13

**Authors:** Yan Liu, Liping Xie, Guihua Gong, Wei Zhang, Baoquan Zhu, Youjia Hu

**Affiliations:** 1 China State Institute of Pharmaceutical Industry, Zhangjiang Institute, Shanghai, China; 2 Shanghai Institute of Pharmaceutical Industry, Shanghai, China; Chinese Academy of Fishery Sciences, China

## Abstract

β-lactam antibiotics are widely used in clinic. Filamentous fungus *Acremonium chrysogenum* is an important industrial fungus for the production of CPC, one of the major precursors of β-lactam antibiotics. Although its fermentation yield has been bred significantly over the past decades, little is known regarding molecular changes between the industrial strain and the wild type strain. This limits the possibility to improve CPC production further by molecular breeding. Comparative transcriptome is a powerful tool to understand the molecular mechanisms of CPC industrial high yield producer compared to wild type. A total of 57 million clean sequencing reads with an average length of 100 bp were generated from Illumina sequencing platform. 22,878 sequences were assembled. Among the assembled unigenes, 9502 were annotated and 1989 annotated sequences were assigned to 121 pathways by searching against the Kyoto Encyclopedia of Genes and Genomes pathway (KEGG) database. Furthermore, we compared the transcriptome differences between a high-yield and a wild-type strain during fermentation. A total of 4329 unigenes with significantly different transcription level were identified, among which 1737 were up-regulated and 2592 were down-regulated. 24 pathways were subsequently determined which involve glycerolipid metabolism, galactose metabolism, and pyrimidine metabolism. We also examined the transcription levels of 18 identified genes, including 11 up-regulated genes and 7 down-regulated genes using reverse transcription quantitative -PCR (RT-qPCR). The results of RT-qPCR were consistent with the Illumina sequencing. In this study, the Illumina sequencing provides the most comprehensive sequences for gene expression profile of *Acremonium chrysogenum* and allows *de novo* transcriptome assembly while lacking genome information. Comparative analysis of RNA-seq data reveals the complexity of the transcriptome in the fermentation of different yield strains. This is an important public information platform which could be used to accelerate the research to improve CPC production in *Acremonium chrysogenum*.

## Introduction

*Acremonium chrysogenum* is an important industrial fungus for the production of cephalosporin C (CPC), one of the major precusors of β-lactam antibiotics. β-lactam antibiotics are the major first line anti-microbial agents. CPC and its semi-synthetic derivatives play important roles in the pharmaceutical industry. In 1948, *Acremonium chrysogenum* was first isolated from Sardinian coastal seawater and was found to produce an antibiotic which inhibited the growth of several Gram-positive and Gram-negative bacteria [1]. Because of the very low yield, the improvement of CPC production level is very important during its industrialization process. Several rounds of mutagenesis were performed and resulted in high production strains with extremely elevated cephalosporin C titers compared to the wild-type strain [2]. These strains are used for industrial production of CPC whose fermentation yield is around 40,000 µg/ml in fed batch cultures.

**Figure 1:**
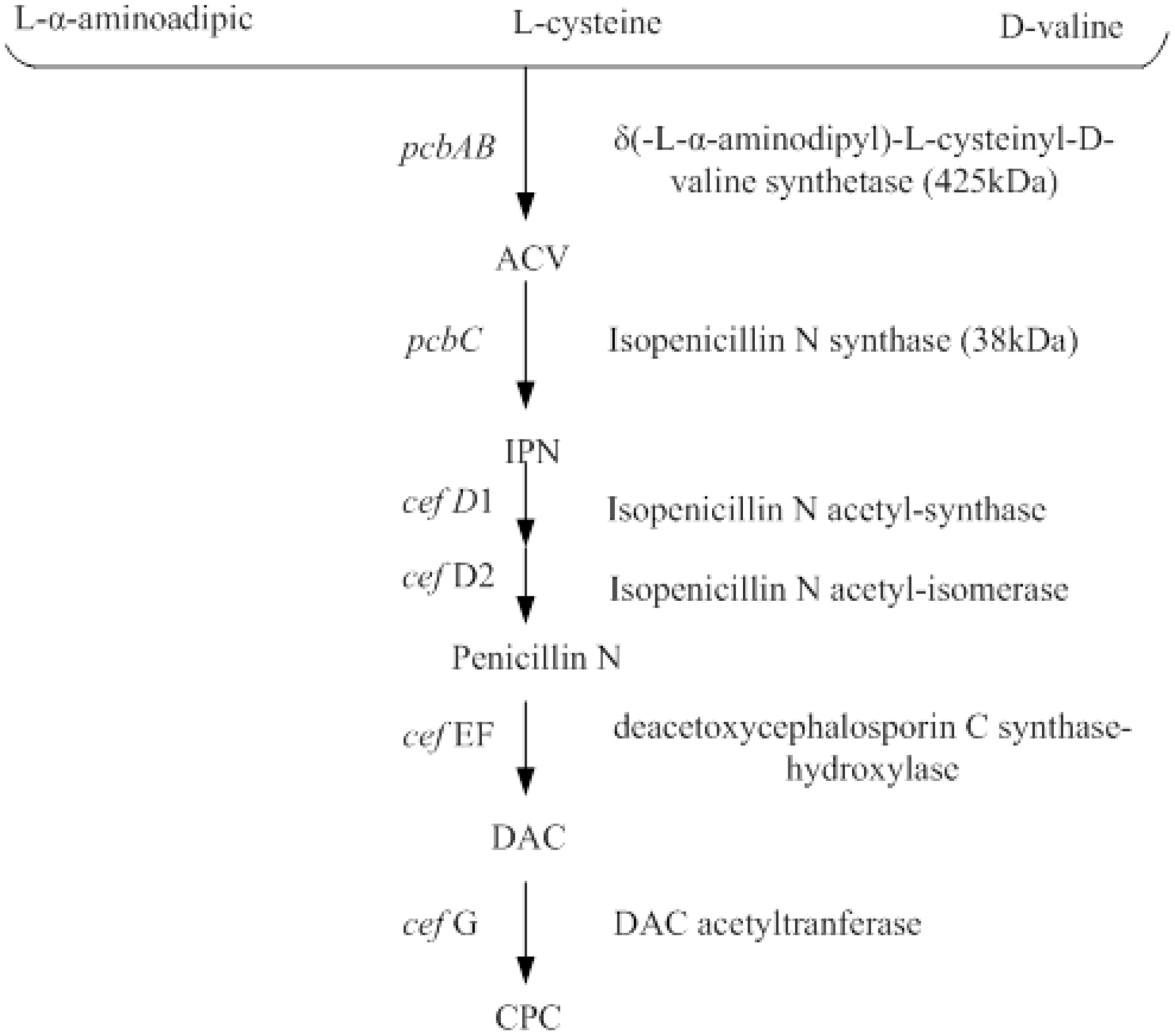
CPC biosynthetic pathway. The schematic representation shows the genes and proteins involved in the biosynthesis of Cephalosporin C. ACV:(-L-α-aminodipyl)-L-cysteinyl-D-valine, IPN:Isopenicillin N, DAC;deactycephalosporin C, CPC:cephalosporin C.

Researchers have studied and reported the biosynthesis and genetic regulation of CPC (Figure 1). The biosynthetic pathway of cephalosporin C in *A. chrysogenum* includes eight enzymatic-catalyzed steps, and the expressions of these enzyme encoding genes are controlled by several regulatory factors (e.g. *CreA*, *PACC*,*CPCR1*) through complex regulatory processes [3], [4]. Recently, there have been several interesting studies on this important fungus investigating its basic physiology as well as potential industrial applications [5], [6]. For example, *CefG* and *cefEF* genes encode the putative potential rate-limiting enzymes [7], [8]. By introducing *cefT* [9], *cefEF* [10], *cefG* [11] and *vgb* [12] genes, CPC production has been improved in *A.chrysogenum* ATCC 11550. *ActrxR*1 encoding the thioredoxin reductase of thioredoxin system is required for normal growth of *A. chrysogenum* and is related with CPC production in methionine supplemented medium [13].

In filamentous fungi, secondary metabolism and morphogenesis are tightly connected processes. In *A. chrysogenum*, AcVEA controls the transcriptional expression of six CPC biosynthesis genes. The *AcveA* disruption strains showed drastic reduction in *cefEF* expression, which encodes the deacetoxycephalosporine/deacetylcephalosporine synthetase [14]. AcVEA is also involved in hyphal fragmentation, which is an active developmental process in the cellular differentiation of *A. chrysogenum* and results in the formation of spherical cells, called arthrospores. Arthrospores are associated with increased antibiotic production, underlining the relationship between secondary metabolism and morphology [15].

Although *A. chrysogenum* industrial strain produces high yield of CPC,little is known about the molecular mechanisms behind how it became such a high CPC overproducer. Furthermore, molecular genetic analysis of this fungus is much more challenging than for other biotechnologically relevant fungi such as *Penicillium chrysogenum* or *Trichoderma reesei* [16], [17]. Although the complete nucleotide sequence of the 27,266 bp mitochondrial genome of the CPC producing fungus *Acremonium chrysogenum* was recently determined using a whole genome shotgun sequencing [18]; this is way not enough for studying strain improvement of *A. chrysogenum* for molecular breeding. Another limitation is the lack of knowledge about the sexual lifecycle and rare conidiospore production in *A. chrysogenum* [15]. These have limited the progress of conducting molecular breeding of *A. chrysogenum*.

Understanding complex functional mechanisms requires the global and comparative analysis of different cellular processes between high-yield (HY) and wild-type (WT) strains. Transcription profiling is the main platform for genome-wide epigenetic analysis. Over the past several years, the next generation sequencing technology has emerged as a cutting edge approach for high-throughput sequence determination [19], [20]. High-throughput RNA-seq technology represents a powerful and cost effective tool for transcription profiling [21] which has enabled investigating the transcriptome for various gene expression studies without the reference of genome sequences [22]. Carrier *et al* identified the candidate genes which could account for lipid over-accumulation, as well as providing insights into the putative life cycle of *Tisochrysis lutea* using RNA-seq approach [23].

In this study, we generated over five giga bases of high-quality DNA sequence with Illumina technology and demonstrated the suitability of short-read sequencing for *de novo* assembly and annotation of genes expressed in a eukaryote without prior knowing of the genome information. Subsequently, we analyzed the transcription levels of 22,878 unigenes, identified the differentially expressed genes between HY and WT strains, some which were validated by using RT-qPCR. The assembled, annotated transcriptome sequences and gene expression profiles provide an invaluable resource for the identification of the relevant genetic modifications produced during the industrial strain improvement program.

## Results

### Comparison of CPC production during fermentation between the HY and WT strain of Acremonium chrysogenum

**Figure 2:**
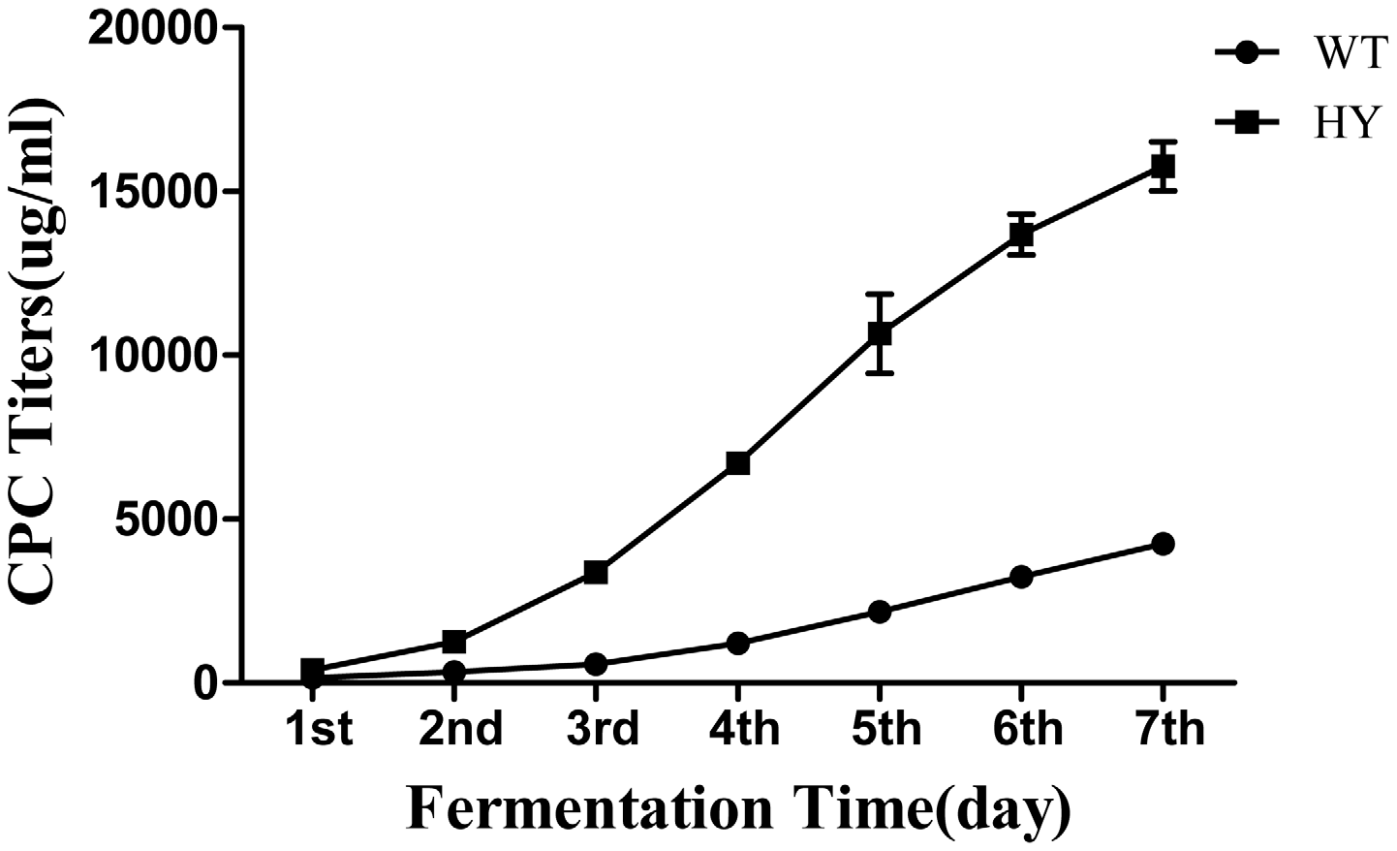
CPC yield between high-yield strain (HY) and wild-yield strain (WT) during the fermentation process. The mean values of three independent experiments with respective standard deviations are shown.

Before isolating the total RNA, we determined their CPC production during fermentation of High-yield (HY) and wild-type (WT) strains of *Acremonium chrysogenum*. Strains were cultured for three consecutive batches. The yield of CPC was recorded every day and the titer curves were drawn accordingly as shown in Figure 2. The CPC production titers of WT and HY were about 4893 µg/ml and 15892 µg/ml, respectively. CPC accumulates continually throughout the fermentation until day 7. Thus, we chose day 7 of the fermentation as the time point to analyze the transcriptome differences between WT and HY.

### Illumina sequencing and reads assembly

**Figure 3:**
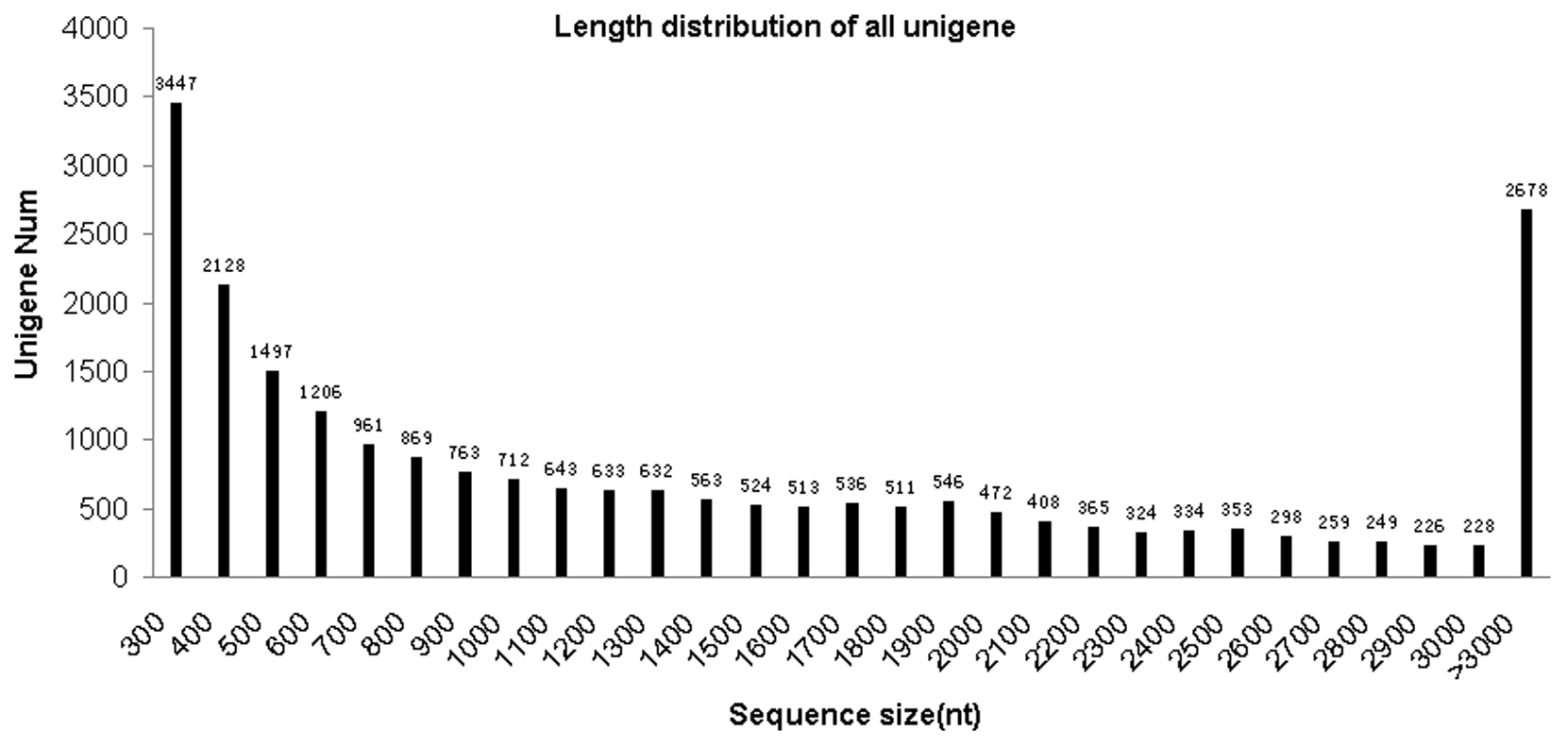
The size distribution of unigenes. The numbers of unigenes matched (with a cut-off E-value of 10^-5^) in NCBI nr databases with different length interval are shown.

To obtain an overview of *Acremonium chrysogenum* gene expression profile in HY and WT strains, two total RNA samples were isolated from the mycelia respectively, and then equal amount of both RNA samples were mixed for Illumina sequencing. An overview of the sequencing and assembly is outlined in Table 1. A total of 57 million clean sequencing reads with an average length of 100 bp were generated. The raw sequencing data was deposited in NIH Short Read Archive (SRA) database and the accession number is SRA169314. To facilitate sequence assembly, we use a software called Trinity to assemble the sequences. A total of 22,878 sequences were assembled, with an average length of 2953 bp. The size distribution of the unigene was shown in Figure 3. Among these sequences, 15,806 were longer than 500 bp, 11,295 were longer than 1000 bp, 5,722 were longer than 2000 bp, and 2,678 were longer than 3000 bp.

**Table 1:** Summary of the sequence assembly after Illumina sequencing.

	Total RNA	HY	WT
Total reads	57217312	21677780	18972520
Total nucleotides	5778948512		
Effective read Numbers	55,733,210		
Data Product (bp)	5,778,948,512		
Average read length	101 bp		
Total all-unigenes	22878		
Mean length of sequences	2953		

Total RNA: mixture of total RNA consisted of RNA from HY and RNA from WT.

### Functional annotation

**Figure 4:**
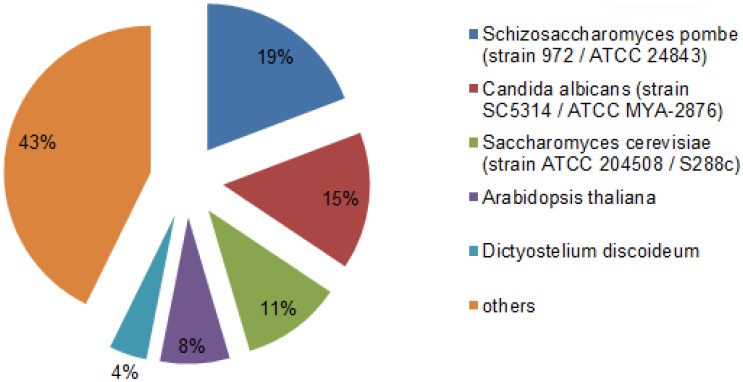
The species distribution among the top hits in the nucleotide database. The number in each inner part shows proportion(%) homologous genes with reference microalgae.

To obtain functional information, gene annotation including homologous protein annotation was performed using gene ontology(GO)and Kyoto Encyclopedia of Genes and Genomes pathway (KEGG) database. All-unigenes sequences were first blasted using BLASTx against the non-redundant NCBI nucleotide database using a cut-off E-value of 10^−5^. The protein functions were predicted from the annotations with the most homologous proteins using the Uniprot and SwissProt database. 9502 annotated uniGenes were obtained by this method. The species distribution among the top hits showed that 19.22% annotated sequences matched with *Schizosaccharomyces pombe* (strain 972/ATCC 24843), followed by *Candida albicans* (strain SC5314/ATCC MYA-2876) (15.17%), *Saccharomyces cerevisiae* (strain ATCC 204508/S288c) (11.05%), *Arabidopsis thaliana* (7.66%), *Dictyostelium discoideum* (4.17%) as shown in Figure 4.

**Figure 5:**
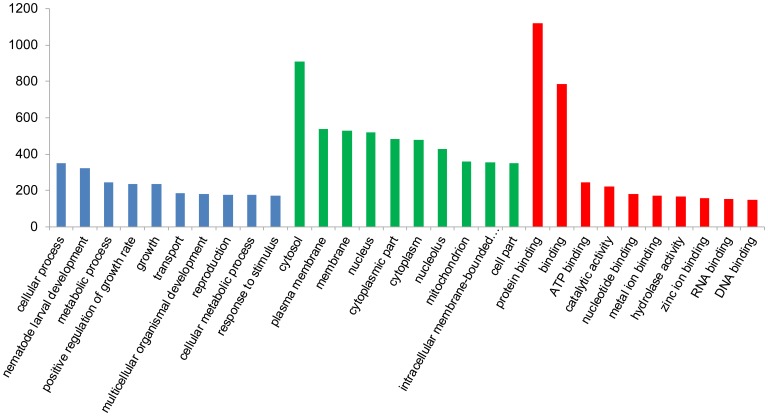
Histogram representation of genes ontology classification. The results are summarized into three main categories: biological process, cellular component and molecular function. The y-axis indicates the number of genes in a category.

**Figure 6:**
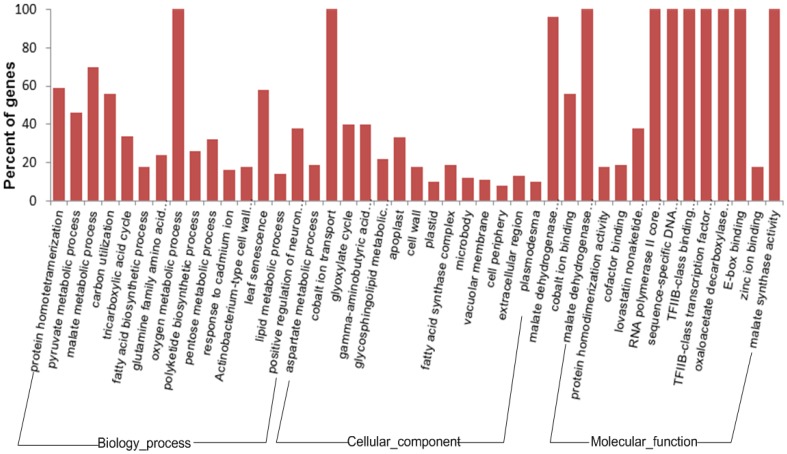
Histogram presentation of differentially expressed Gene Ontology classification. The results are summarized in three main categories: biological process, cellular component, and molecular function. The y-axis indicates the percentage of a specific category of genes in the main category.

**Figure 7:**
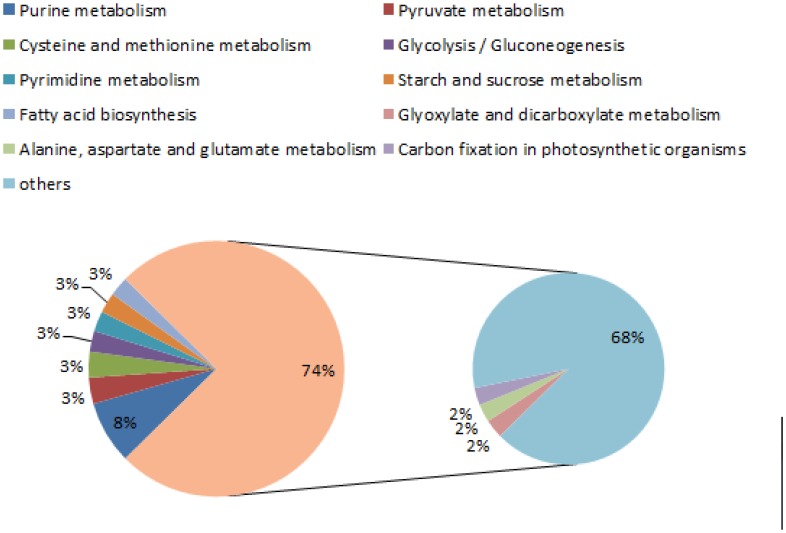
Pathway classification assigned to annotated genes. Unigenes were annotated by KEGG database.

Gene Ontology(GO)is an international standardized gene functional classification system, which fully describes the properties of genes and gene products in an organism. GO was analyzed using Blast2go software (http://www.blast2go.com/b2ghome). Based on sequence homology, those sequences were categorized into functional groups including “Biological process” (a total of 5279 sequences), “Cellular component” (altogether 5132 sequences) and “Molecular function” (5032 sequences in total, see Figure 5). Among these three main categories, ‘Cellular progress’, ‘Cytosol’ and ‘Protein binding’ were the three major sub-categories. A high percentage of genes came from categories of ‘Metabolic process’, ‘Regulation of growth rate’, ‘Cytoplasmic part’, ‘Catalytic’, and ‘ATP binding’. To further identify the different gene expression levels during fermentation (WT vs. HY), the GO functional analysis was carried out on the differentially expressed genes (Figure 6). To identify the biological pathways that are active during the fermentation of *Acremonium chrysogenum*, 9502 annotated sequences were mapped to the reference canonical pathways in KEGG. 1989 sequences were assigned to 121 KEGG pathways (Figure 7). The pathways with most occurring frequency among the annotated sequences were Purine metabolism (158 members); Pyruvate metabolism (66 members) and Cysteine and methionine metabolism (65 members), which were related with energy metabolism and precursor biosynthesis in CPC biosynthesis. Furthermore, there were also some other metabolism related proteins (Glycolysis/Gluconeogenesis, Pyrimidine metabolism). These information provide a valuable resource for investigating specific processes, functions and pathways during the CPC fermentation of *Acremonium chrysogenum*.

### Changes in gene transcription profile during fermentation of HY and WT strains

After comparing the transcriptional levels of HY and WT strains, a total of 4329 unigenes with significantly different expression levels were identified, Among the differentially expressed genes, ‘Oxygen metabolism’, ‘Formate dehydrogenase’, ‘NADP+ oxidoreductase’ activity were predicted to strongly correlate with the metabolism of CPC. Among these 4329 unigenes, 1737 were up-regulated and 2592 were down-regulated. We found that more than 70% of these unigenes have no homologous sequences in the NCBI database. These may be uniquely expressed genes by *Acremonium chrysogenum* without presenting in other species.

### Functional annotation of differentially expressed genes

To understand the functions of differentially expressed genes, we chose q value ≤0.05 as significant among the differentially expressed genes to map the related pathways in KEGG database. In total, among all the genes with KEGG pathway annotation, 48 differentially expressed genes were identified in 24 significant pathways, which were involved in Glycerolipid metabolism, Galactose metabolism, and Pyrimidine metabolism. Notably, specific significance of genes was observed in pathways associated with energy and lipid metabolism, such as Fatty acid metabolism, Metabolism of xenobiotics by a putative cytochrome P450, Nitrogen metabolism, and Degradation of CPC biosynthetic precursors including valine, leucine, isoleucine and lysine.

### RT-qPCR validation

To confirm that the differentially expressed unigenes obtained from Illumina sequencing and computational analysis were truly transcribed at different levels during fermentation between HY and WT strains, 11 up-regulated genes and 7 down-regulated genes related to the CPC synthesis were chosen for RT-qPCR assays (Table 2).

**Table 2:** The relative expression of gene between HY and WT strains transcriptome.

Gene_id	Description	q-value	Log2(HY_RPKM/WT_RPKM)
***Carbohydrate metabolism and energy***			
comp1129	Putative NADPH dehydrogenase	0.0037	4.42
Comp1270	Carbonyl reductase	0.0287	2.85
Comp8269	β-glucosidase	0.0121	−2.15
comp2106	Alpha-galactosidase	0.0000832	−3.2
Comp2667	Acyl-CoA carboxylase	0.00201	3.47
Comp5940	Acyl-CoA desaturase	0.0021	2.62
Comp1839	Methyltransferase	0.000828	−3.04
Comp7031	Polyamine transporter	0.00001	3.63
comp4981	Potential zinc-binding dehydrogenase	0.00004	6.57
***CPC biosynthesis precursors***			
Comp1533	Cystathionine gamma-lyase	0.00533	3.43
Comp1669	NRPS	0.00003	5.42
Comp16936	Acetylornithine aminotransferase	0.0005	−5.07
***Oxidative stress***			
Comp1988	Cytochrome c oxidase	0.0182	−2.18
Comp1893	Peroxisomal membrane protein	0.0056	−2.31
Comp782	Thioredoxin reductase	0.0000000014	−1.85
***Other secondary metabolites***			
Comp7927	Lovastatin nonaketide synthase	0.0001	3.45
***Defense and virulence mechanisms***			
comp5590	Flavin-containing monoxygenase	0.000734	2.70
Comp4194	PRY1	0.00344	2.48

Bold letters indicate metabolic pathways in which the genes are involved.

**Figure 8:**
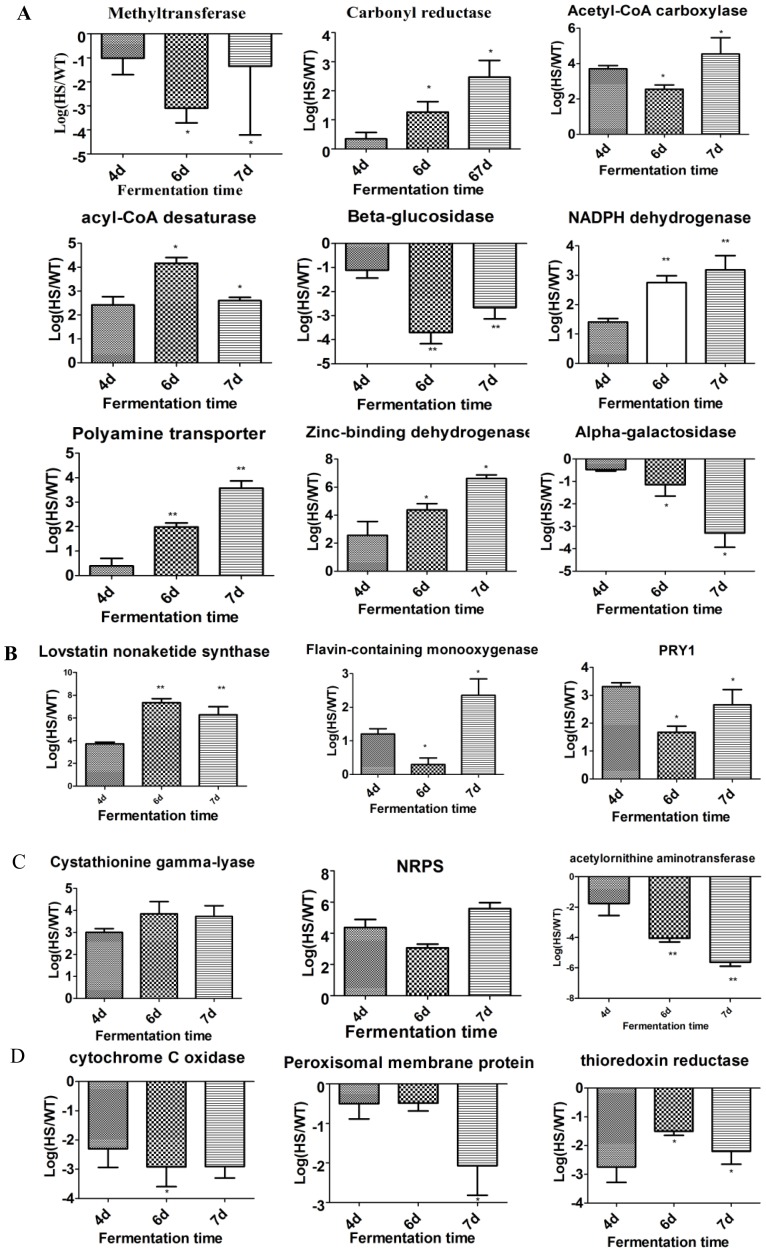
RT-qPCR analysis of the transcriptional levels of 18 unigenes between HY and WT strains during fermentation. A. related Carbohydrate metabolism and energy genes. B. Other Secondary Metabolites and Defense and virulence mechanisms genes, C. CPC biosynthetic precursor associated genes. D. Related Oxidative stress defenses genes. The expression was analyzed at days 4, 6 and 7. Error bars represent ±SD. Asterisk represent statistically significant differences between the expression levels of the genes (statistics were generated based on day 4 using student t-test and p<0.05 was account as significant difference).

To analyze the gene transcription of HY and WT strain, the transcription level of WT was set as the reference standard. The results showed that the selected genes expressed different patterns in day 4, day 6 and day 7 of fermentation. Among them, seven genes related to Carbohydrate Metabolism and Energy were validated. In HY strain, compared to WT strain, a putative NADPH a putative dehydrogenase, a putative carbonyl reductase, a putative aceyl-CoA carboxylase, a putative acyl-CoA desaturase were up-regulated, while a putative alpha-galactosidase, a putative β-glucosidase and a putative methyltranserase were down-regulated. Furthermore, the genes involved in CPC biosynthetic precursors were also validated (a putative Cystathionine β-lyase, a putative NRPS were up-regulated and a putative Acethylonithine aminotransferase was down-regulated in HY strain). Some genes related to other secondary metabolites such as a probable Lovastatin nonaketide synthase (up-regulated) and Defense and virulence mechanism components such as a probable Flavin-containing monooxygense and a probable PRY1 (up-regulated) also showed different transcription levels between HY and WT strains. In addition, oxidative stress defenses were closely related to secondary metabolites, including a hypothetical a hypothetical cytochrome oxidase, a hypothetical peroxisomal membrane protein and thioredoxin reductase which were down-regulated in HY strain. Results are shown in Figure 8. Overall, the results of RT-qPCR were consistent with the Illumina sequencing. Although these genes were assigned to different metabolic pathway, they were very likely related to CPC biosynthesis.

## Discussion

High-throughput mRNA sequencing technology is a fast, efficient, and cost-effective way to characterize the transcriptome. It is especially suitable for gene expression profiling in non-model organisms without prior genome annotation [22]. In this study, we applied RNA-seq technology, based on the Illumina HiSeq™ 2000 platform and obtained 57,217,312 clean sequencing reads (average length >100 bp) by de novo assembly. Among them, 22,878 assembled unigenes were successfully annotated. Between the transcriptomes of HY and WT CPC producers, approximately 4329 unigenes were found to be expressed at significantly different levels. The present study provided good comprehensive genetic and genomic information for *Acremonium chrysogenum*.

RT-qPCR results were consistent with the Illumina sequencing. In 18 tested unigenes, 11unigenes were up-regulated, 7 unigenes were down-regulated. The findings were classified as follows:

### Carbohydrate Metabolism and Energy

In this study, a putative NADPH dehydrogenases, which are abundant in the respiratory chain, were found to be up-regulated. None of these additional electron-transport enzymes pump protons and therefore do not contribute to energy conservation via ATP synthesis. In *Acremonium chrysogenum*, there is an uncoupling protein that bypasses the ATP synthase reaction [24]. Another putative protein, Zinc-binding dehydrogenase, was markedly elevated in HY compared with WT strains. Zinc-binding dehydrogenase is highly homologous with NADPH quinone oxidoreductase [25], which may be capable of oxidizing externally added NADPH. In fermentative yeasts, alternative NADH: ubiquinone oxidoreductases have acquired a special function that is preferentially metabolized via anaerobic glycolysis and ethanolic fermentation [26]. Therefore, a putative Zinc-binding dehydrogenase might convert the other substrate to NAD(P)H. An increase of NADPH levels has been proved to be strongly correlated with β-lactam production [27], [28]. A putative β-glucosidase was overrepresented in the WT strain. This enzyme is involved in cellulose degradation [29], which indicates the ability of the WT to utilize cellulose as a carbon source. A probable carbonyl reductase is overexpressed in HY strain. Kataoka *et al* reported that carbonyl reductase coupled with an NADPH regenerating system has the advantage for the synthesis of (R)-CHBE [30]. Alpha-galactosidase belongs to a family of glycosylhydroxlases, for degradation of glycosidic bonds in complex sugars such as galactose oligosaccharides, galactomannans, and galactolipids, and therefore directly involved in the metabolism [31]. Acyl-CoA carboxylase is a biotin-dependent enzyme that catalyzes the irreversible carboxylation of acetyl-CoA to produce malonyl-CoA, subsequently synthesize fatty acid, which responsible for the initial steps of CPC biosynthesis [32]. Another gene related to fatty acid biosynthesis is acyl-CoA desaturase, which can utilize O_2_ and electrons from the reduced cytochrome b5 domain to catalyze the insertion of a double bond into a spectrum of fatty acyl-CoA substrates. In yeast, the degree of acyl chain desaturation was modulated by the acyl-CoA desaturase gene expression [33]. A putative acyl-CoA carboxylase and a probable acyl-CoA desaturase genes were over expressed in HY strains. They may promote the fatty acid biosynthesis, accelerate energy metabolism, and thus augment the biosynthesis of CPC.

Polyamines are essential for cell growth, which are regulated by biosynthesis, degradation and transportation. Polyamine transporter (*DUR3*) is a polyamine-preferential transporter. The cell growth was inhibited by the disruption of *DUR3* and *SAM3* [34]. The increased expression of a hypothetical *DUR3* gene in HY compared with that in WT may be related to the growth of *Acremonium chrysogenum* and contribute to the high production of CPC in HY.

### Other Secondary Metabolites

Lovastatin, a fungal secondary metabolite capable of lowering the cholesterol level in blood, is synthesized by two polyketide synthases and several modifying enzymes. Lovastatin nonaketide synthase was found to be up-regulated. Synthesis of the main nonaketide-derived skeleton requires the lovastatin nonaketide synthase [35]. Therefore this probable protein may be required to form the skeleton of CPC biosynthesis. Although this protein is likely involved in lovastatin biosynthesis, the improved expression of this probable gene in the HY strain compared with WT strains may be one of the mechanisms that contribute to increased CPC titers in the HY strain and it may be indirectly related to CPC biosynthesis.

### Defense and virulence mechanisms

The flavin-containing monooxygenases (FMOs) consist of a group of enzymes that catalyze chemical reactions via the bound cofactor flavin. They contain one molecule of FAD per monomer. The main function of FMO is to add one molecule of oxygen to lipophilic compounds, making them soluble to ensure rapid excretion [36]. HY strains showed overrepresentation of a putative FMO when compared with WT strain. Therefore, we presume that this probable gene stimulated the secretion of CPC. PRY1 is a member of sterol-binding protein, belonging to the CAP protein superfamily, which is essential and sufficient for lipid export and sterol binding [37]. The CAP protein plays a significant role in the fungal infections of avocado and tomato fruits [38]. The high expression of a probable PRY1 gene in HY may prevent fungal infections and prolong the life span of mycelium, which benefit the CPC biosynthesis.

### CPC biosynthetic precursor amino acids

The non-ribosomal condensation of the three precursor amino acids (L-α-aminoadipic acid, L-cysteine, and L-valine) is the initial step of CPC biosynthesis. Some other genes were involved in metabolism of amino acid precursors for CPC biosynthesis in HY and WT strains. A putative Cystathionine β-lyase, an enzyme involved in the biosynthesis of cysteine from methionine by transculturation [39], was up-regulated in HY. This indicated that the HY had two improved cysteine biosynthetic pathways. In addition, a probable branched-chain amino acid aminotransferase, was overexpressed in HY. This protein has been reported to participate in the metabolism of valine and serine [40]. A probable acetylornithine aminotransferase, required for arginine biosynthesis [41], was down-regulated in HY. Improved arginine biosynthesis can affect valine and serine biosynthesis, subsequently affect the CPC biosynthesis.

The generation of modified amino acid precursors for incorporation in nonribosomal peptide synthesis(NRPS) plays a crucial role in the generation of peptide natural products [42]. In this study, the transcription of a putative NRPS in HY was much higher than that in WT, suggesting that putative NRPS stimulated the biosynthesis of precursors of cephalosporin C.

### Oxidative stress defenses

Three oxygen-consuming steps have been identified in the pathway of CPC biosynthesis, and dissolved oxygen content is considered to be of the most important factors affecting the production of CPC [43], [44]. Extra oxygen is potentially toxic for fungal growth and other cell processes. Long-term oxidative stress can cause genome-wide transcriptional and proteome-wide translational changes in *Aspergillus nidulans* [45]. Oxidative stress could stimulate the onset of secondary metabolic biosynthesis in fungi. Accumulation of deoxynivalenol and its 15-acetylated form is significantly modulated by oxidative stress in *Fusarium graminearum* [46]. Oxidative stress is also involved in the regulation of OTA biosynthesis in *Aspergillus ochraceus* [47].

In addition, the natural byproducts of mitochondrial respiration and oxygen consumption were noted to be converted to reactive oxygen species (ROS), such as superoxide anion radicals. The formation of ROS involves receiving a single electron from oxygen by important enzymes such as cytochrome C oxidase. Loss of cytochrome C oxidase promotes RAS-dependent ROS production [48], [49], and peroxisomal membrane protein production [50]. In HY, the transcription level of a probable cytochrome C oxidase and a probable peroxisomal membrane protein are lower than that in WT strain. This may result the reduction of oxygen consumption, thus enhancing the biosynthesis of CPC.

The thioredoxin system is used for oxidative stress defenses in fungi. Thioredoxin reductase (TrxR, a member of the thioredoxin system)-encoding gene (*ActrxR1*) contains an FAD binding domain, a redox domain, and an NADPH binding domain. Disruption of *ActrxR1* in *A.chrysogenum* led to the formation of smaller colonies and hyphal swelling in Tryptic soy agar. The *ActrxR1* disruption mutant grew normally, but its CPC production increased [13]. In HY strain, the transcription level of thioredoxin reductase is lower than that in WT strain suggesting that thioredoxin reductase plays a role in the negative regulation of CPC biosynthesis.

**Figure 9:**
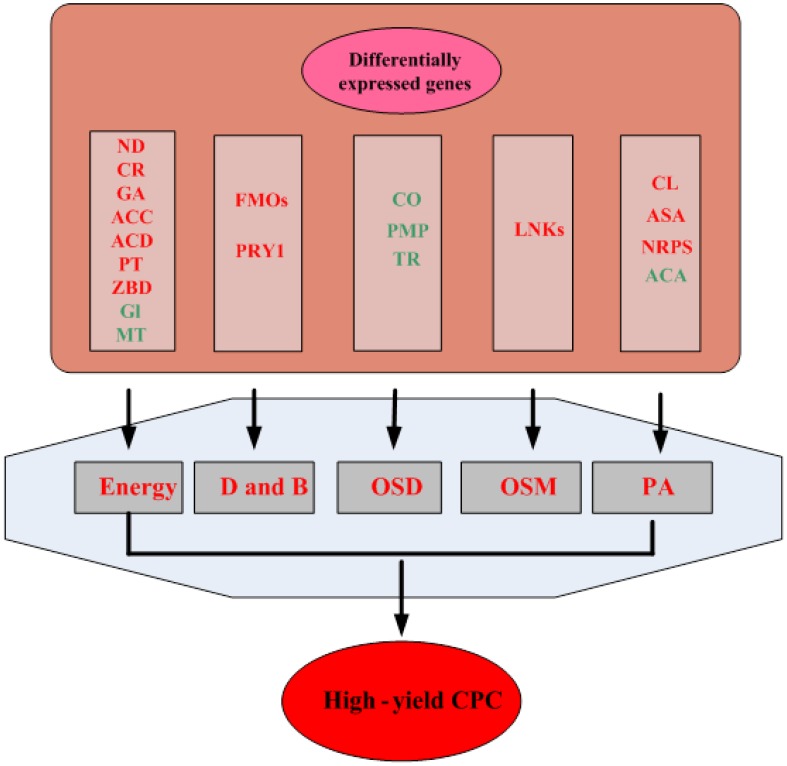
Preliminary model of HY strain vs. WT strain according to the results obtained from comparative transcriptome. Red color represents up-regulated genes, and green color represents down-regulated genes. ND, NADPH dehydrogenase; CR, carbonyl reductase; GA, Alpha-galactosidase; ACC, acyl-CoA carboxylase; ACD, acyl-CoA desaturase; PT, polyamine transporter; ZBD, Zinc-binding dehydrogenase; Gl,β-glucosidase; MT, Methyltransferase; FMOs, flavin-containing monooxygenase; CO, cytochrome oxidase; PMP, peroxisomal membrane protein; TR,Thioredoxin reductase; LNKS, Lovastatin nonaketide synthase; CL, Cystathionine β-lyase. ASA, Asparate aminotransferase; ACA, Acethylonithine aminotransferase. D and B, Defense and virulence mechanisms; OSD, Oxidative stress defenses; OSM, Other Secondary Metabolites; PA, CPC biosynthetic precursor acids.

In short, a global view of the genes differentially expressed in HY strain compared with WT strain revealed that the improvement of CPC yield was a complex system, which involved in energy metabolism, CPC biosynthetic precursors acids, oxidative stress defense and other metabolisms as well(Figure 9).

## Conclusions

In the present study, by using Illumina sequencing, we obtained transcriptome data of *A.chrysogenum*, assembled 22,878 unigenes, and annotated 9502 of those unigenes. Comparing the two transcriptomes of HY and WT strains, a total of 4329 unigenes expressed at significantly different level were identified, among which 1737 were up-regulated and 2592 were down-regulated. The differentially expressed genes between HY and WT strain can be targets for further study on the gene expression, genomics, and functional genomics of *A. Chrysogenum*.

## Material and Methods

### Strains and culture conditions

The HY strain *A.chrysogenum* 84-3-81-41 (CPC production is higher than 15 mg/ml) and the WT strain *A.chrysogenum* ATCC 11550 (CPC production is around 4 mg/ml) were grown on agar medium [5]. Appropriate amount of spores from slant culture of *A. chrysogenum* was inoculated into 20 ml seed medium (corn steep liquor 3%, sucrose 3.5%, glucose 0.5%, methionine 0.05%, (NH_4_)_2_SO_4_ 0.8%, CaCO_3_ 0.5%, bean oil 1%, pH 6.5) and incubated for 3 days at 28°C, 230 rpm. Then, 2 ml seed culture was inoculated into 20 ml fermentation medium (corn steep liquor 10%, starch 3%, dextrin 6%, glucose 0.5%, methionine 0.6%, urine 0.3%, KH_2_PO_4_ 0.9%, MgSO_4_ 0.3%, (NH_4_)_2_SO_4_ 1.3%, CaCO_3_ 1%, trace element 1%, bean oil 2%, pH 6.2) and fermentation was carried out for 7 days at 25°C. Mycelium were collected by centrifugation at 10,000 rpm, washed twice with ddH_2_O, and store at −80°C.

### RNA Extraction and cDNA library preparation

Mycelium were collected by centrifugation followed by grinding in liquid nitrogen. Total RNA was then isolated by using UNIQ-10 Column Trizol Extraction Total RNA kit (Sangon, Shanghai) according to the manufacturer's instruction. Total RNA was purified by the Qiagen RNeasy mini kit (Qiagen, velencia, CA). Purified RNA was analyzed on a 2100-Bioanalyzer (Agilent Technologies, Santa Clara, CA) to determine the quantity. The purity and integrity of RNA samples were estimated by 260/280 ratio (above 2.0) and RIN (RNA integrity number, greater than 7.0). The RNA samples were then digested by DNaseI for 30 min at 37°C to remove potential genomic DNA. The integrity was further evaluated by electrophoresis on ethidium bromide-stained 1.0% agarose gels. Obtained RNA was dissolved in 20 µl DEPC-H_2_O and stored at −70°C. High quality total RNA (1 µg) was used as the starting material for sequencing. The Truseq RNA sample preparation kit was used for the mRNA purification and fragmentation, and the RNA fragments were used as templates. First-strand cDNA was synthesized by using random hexamer as primers. Second-strand DNA synthesis was carried out using DNA polymerase I, dNTPs and RNase H. Then double strands cDNA was then purified for end repairing, dA tailing, adaptors ligation and DNA fragments enrichment. Resulting library size was checked by using DNA specific chip such as the Agilent DNA-1000 on Agilent Technologies 2100 Bioanalyzer. cDNA libraries were quantified by using Qubit (invitrogen, CA, USA) according to the Qubit user Guide. The total RNA was fragmented into small pieces using divalent cations under elevated temperature.

### Analysis of Illumina sequencing results

The total cDNA samples were sequenced using the Hi-SeqTM2000 platform (Illumina). The raw data was deposited into NIH SRA database under accession number of SRA169314 (NCBI). After raw data filtering, clean reads were acquired for the analysis. *De novo* assembly of transcriptome was performed by a short read assembly program called Trinity [51]. First, with certain length of overlap, Trinity formed longer fragments without N. These longer sequences were analyzed using sequence clustering software [52]. These sequences were defined as unigenes, and further sequence splicing and redundancy removal were performed to acquire non-redundant unigenes.

For further analysis, blastx (BLAST, the basic local alignment search tool) alignment (E value<10^−5^) was performed against various protein databases including Swiss-Prot, KEGG (Kyoto Encyclopedia of Genes and Genomes). Unigene annotations provide information regarding to the expression and functional annotation of the identified genes. The differentially expressed genes were defined as those with false discovery rate (FDR) ≤0.001 and ratio of reads per kilobase per million (RPKMs) >2. Using Blast2GO program we obtained GO annotation of the unigenes. Subsequently, we used WEGO software to carry out GO functional classification for all-unigenes and distribution of gene functions of species at macro level. The KEGG pathways was carried out according to KEGG(http://www.genome.jp/kegg/) database [53]. For gene expression levels between HY and WT strains analysis, genes were filtered according to criteria described above (at least a two-fold change and q value< = 0.05).

### RT–quantitative PCR

The transcriptional level of candidate genes was determined by RT-qPCR. Gene specific primers were designed using Primer Premier 5.0 software, (the primers used for RT-qPCR analyses are listed in Table S1). The testing strains were grown in the fermentation culture for 4 and 7days. Total RNA were then extracted according to above method. 1 µg of total RNA was reverse transcribed to cDNA using a PrimeScripTM RT reagent Kit (TaKaRa) according to the manufacturer's instructions. A total of 21 genes were chosen for RT-qPCR. The house-keeping gene actin (GenBank accession number AF056976) was used as the internal control. RT-qPCR was performed in Rotor-Gene 3000 (Corbett) under certain conditions:10 min hold at 95°C followed by 40 cycles of denaturation at 95°C for 10 s, annealing and elongation at 65°C for 40 s. Fluorescence signals were collected at each annealing and elongation step. Three flask samples were cultured for each strain and triplicates were used in RT-qPCR. The relative transcriptional levels were calculated from cycle threshold values using the △△CT method.

## Supporting Information

Table S1 **The sequences of primers for RT-qPCR analysis.**
(DOC)
