# Supplementary material for: De novo Comparative Transcriptome Analysis of Acremonium chrysogenum: High-Yield and Wild-Type Strains of Cephalosporin C Producer
Source: PLoS One. 2014 Aug 13;9(8):e104542. doi: 10.1371/journal.pone.0104542 (PMC4131913; doi:10.1371/journal.pone.0104542)
Supplement: Table S1 — The sequences of primers for RT-qPCR analysis. (DOC) [file pone.0104542.s001.doc]

**Table S1: The sequences of** primers for RT-qPCR analysis

| Genes | Name of primer | sequence |
| --- | --- | --- |
| **Up-regulated genes** | | |
| NADPH dehydrogenase | Sense  Anti-sense | GCCCCCAATTCCCCAAAGAT  ACTTTGTCCTCAACGCGGCG |
| Carbonyl reductase | Sense  Anti-sense | AGGCCAGTTTTGGCACTCCGATAG  TATTCTTGGCCAGTGGCGCG |
| NRPS | Sense  Anti-sense | CCCAAGCCATTCATCCAGCA  GCCCTTTGAACAACCTGGACAAGT |
| Lovastatin nonaketide synthase | Sense  Anti-sense | GGTAGGTCATTAAGACACGGATCCG  GCAACATCGCGATCAAATTAAGGC |
| Flavin-containing monoxygenase | Sense  Anti-sense | ATGGCGGCAGAACAACACCA  CAGCGTGCGGATGAGGTTGA |
| Acyl-CoA dehydrogenase | Sense  Anti-sense | AGGCATTCCCTGATGGCACAA  GGTGGTGTTGAGCCAGTTGATGTG |
| Acyl-CoA carboxylase | Sense  Anti-sense | ACGGGCATCACCCTCAGAACTTC  AGACAGAGAGGAAGGCGATCGG |
| PRY1 | Sense  Anti-sense | CAGAGGTCGATGCTCTCCGTCA  GAAGCAAACAACTACCTCCGCAAC |
| Polyamine transporter | Sense  Anti-sense | CGAAGAAGCGGTACTGCTGGCT  TCCCTTTGCCCAGCAACTGG |
| Zinc-binding dehydrogenase | Sense  Anti-sense | CAGCTGCCCGTACTTCTCGTCA  CCGTCATGGCGTCGTGTTGT |
| Asparateaminotranferase | Sense  Anti-sense | TCAGCCCGGGCGTTAGAGAGTA  TCTGCACGAGCGACGACGTT |
| Lystathionine gamma-lyase | Sense  Anti-sense | GCGCGGAGCACGTCATTCTT  TGCCCAGCAGCATGACCCAT |
| **Down-regulated genes** | | |
| Alpha-galactosidase | Sense  Anti-sense | CTGGTACTCGAGCAGCTCGAATCC  GAATCGGGGAGGTTGCTTGTTG |
| Alcohol dehydrogenase | Sense  Anti-sense | TTGCAGATCTACCTATGATGGGCG  CCGACGAGCTGTCCTATGAGCA |
| Malate synthase | Sense  Anti-sense | GGCCAGCACCGAAACCATTC  GGCTTTCCGGGTAGGGTTGAAG |
| Methyltransferase | Sense  Anti-sense | ATGGTCGCCTCATCAACTGCTTC  GCCTCCTTGTTCTTGGACAGCATG |
| Acetylonithine aminotransferase | Sense  Anti-sense | CGATACTTCTTGTCCAGTTCGAGCC  CATTGAAAGAGCCCATACCAGCATA |
| Beta-glucosidase | Sense  Anti-sense | ATAAATTCTCTCAGGAGAAGGGCCG  CGGGTTATTTTGCGTGGTCTCTTA |
| Thioredoxin reductase | Sense  Anti-sense | CCAGATCGGCAAGGTACTTTTCTG  TCATCACCAAGCCTGGCACCAC |
| Cytochrome c oxidase | Sense  Anti-sense | CTGCAGCTGCTTCAGCTTCTTCTG  ATCTACATGGTCCCCTTGCCCC |
| Peroxisomal membrane protein | Sense  Anti-sense | CACGAAGGGTGCAAGGTGTCTG  GACTGGTGAAGCGCAAAGCG |
